# Correlation between Obstructive Sleep Apnea and Left Ventricular Diastolic Function Assessed by Echocardiography

**DOI:** 10.36660/abc.20190695

**Published:** 2019-12

**Authors:** Cláudio L. Pereira da Cunha

**Affiliations:** Universidade Federal do Paraná, Curitiba, PR - Brazil

**Keywords:** Cardiovascular Diseases, Sleep Apnea,Obstructive, Hypertrophy,Left Ventricular, Indicators of Mortality and Morbidity, Heart Failure, Echocardiography/methods, Polysomngraphy/methods, Risk Factors.

Obstructive sleeP apnea (OSA) is a disease characterized by recurrent upper airway obstruction during sleeP , resulting from the repetitive collapse of these pathways, resulting in hypoxia and sleeP fragmentation.^1^ It is a very common disorder, being more common in men, but it can also affect women and children.^1^ Its prevalence has been estimated at approximately 14% among men and 5% among women and OSA has been defined in these studies as the presence of an apnea-hypopnea index > 5 events per hour of sleeP , associated with 4% of oxygen desaturation.^2^

OSA is associated with a significant increase in sympathetic activity during sleeP , influencing heart rate and blood pressure. The increase in the sympathetic activity is induced by a number of mechanisms, including chemoreflex stimulation by hypoxia and hypercapnia, baroreflex, endothelial dysfunction, and venous return and cardiac output alterations.^3^

The abnormal pattern of breathing during sleeP , associated with repeated awakenings, results in hemodynamic, autonomic, inflammatory, and metabolic effects that may contribute to the pathogenesis of several cardiovascular diseases: systemic arterial hypertension, coronary disease, cardiac arrhythmias (atrial fibrillation, or sudden death due to arrhythmia), heart failure, left ventricular (LV) hypertrophy, cerebrovascular accident, and pulmonary hypertension.^4^

OSA should be suspected whenever a patient presents with excessive daytime drowsiness, snoring and asphyxiation during sleeP , particularly in the presence of risk factors such as obesity, male gender and older age. However, OSA is not a clinical diagnosis and objective tests should be performed for the diagnosis.^5^

In this issue of the Brazilian Archives of Cardiology, Leite et al.^6^ show the correlation between the risk of OSA and echocardiographic parameters related to LV diastolic dysfunction. A total of 354 individuals included in the study answered the Berlin Questionnaire (BQ), a tool used to estimate the risk of OSA, with 63% of them being classified as having a high risk for this disorder.

Most sleeP disorder researchers do not recommend the routine use of assessment tools such as questionnaires or algorithms to select patients at higher risk for OSA, as these tools have not shown to be superior to the clinical history and physical examination in the clinical assessment of these patients.^7^

The American Academy of SleeP Medicine clinical guideline published in 2017 strongly recommends that questionnaires and clinical prediction algorithms should not be used to diagnose OSA in the absence of polysomnography.^7^ These tools are considered to be of low diagnostic accuracy. Regarding the QB, used in the aforementioned study in this issue,^6^ the literature discloses a large number of false negative results, thus limiting its usefulness as a tool for OSA diagnosis. A review of 19 studies that analyzed the performance of the BQ compared to polysomnography data showed an overall sensitivity of 0.76 (95% CI: 0.72 to 0.80), whereas the overall specificity was 0.45 (95% CI: 0.34 to 0.56). This result discloses a very high number of false negative results (209 of 1,000 patients), with compromised diagnostic accuracy.

Therefore, this is a limitation of the study under analysis, since we do not study a population of OSA patients, but individuals at high risk of OSA, using a diagnostic tool considered to be of low diagnostic accuracy.

It is understood that, in an environment without sleeP disorder specialists, the assessment tools, such as questionnaires and clinical prediction algorithms, may be useful because they promote the uniformity of sleeP assessment, and, when necessary, expand their use counting on other health team professionals to apply them. However, one should bear in mind that the application of these tests does not replace a good clinical evaluation, with anamnesis and physical examination, much less the polysomnography, which remains the gold standard for the diagnosis of OSA.^7^

The study by Leite et al.^6^ aimed to evaluate the behavior of echocardiographic parameters in OSA. Restrictions are made to the characterization of the studied population (patients at risk of OSA according to the BQ), but the results obtained were consistent with the literature findings. Increased left atrial volume and the behavior of mitral flow indices characterize LV diastolic dysfunction.^6^

Left atrial enlargement in OSA was characterized in a recent study by Cetin et al.,^8^ who analyzed 55 patients diagnosed with OSA through polysomnography. Left atrial volume and left atrial deformation parameters were assessed through speckle-tracking echocardiography (strain and strain rate). Exercise capacity was also assessed through exercise testing. It was concluded that LV diastolic dysfunction is more prevalent in patients with severe OSA and is associated with reduced exercise performance. Left atrial remodeling contributed to exercise capacity prediction in this subgrouP of patients.^8^

A meta-analysis of 17 studies on LV remodeling and dysfunction in OSA,^9^ concluded that this syndrome leads to left atrial dilation, and LV hypertrophy, dilation, increased mass and systolic function reduction.^9^ The treatment of OSA may be beneficial in preserving LV structure and function.^9^

An interesting review carried out in Romania by Sascau et al.,^10^ demonstrates that the moderate and severe forms of OSA are associated with increased atrial volumes, altered LV diastolic function and then LV systolic function. The assessment of right ventricular ejection fraction may also be compromised, being better evaluated by three-dimensional echocardiography. Moreover, the contribution of two-dimensional speckle-tracking echocardiography has been very effective, differentiating between active and passive wall movements. Abnormal strain values, a subclinical marker of myocardial dysfunction, can be detected even in patients with normal ejection fraction and volumes. LV longitudinal strain is more affected by the presence of OSA.^10^

In conclusion, the work by Leite et al.^6^ highlights the contribution of echocardiography in OSA evaluation, a frequent disorder with different facets of pathophysiological interaction with cardiovascular diseases. The technological development of echocardiography, particularly with three-dimensional and speckle tracking techniques, shows a continuing contribution to the study of OSA.
